# Assessment of Antimicrobial Stewardship practices using process measures in a Nigerian tertiary hospital: a retrospective study

**DOI:** 10.1017/ash.2026.10343

**Published:** 2026-03-27

**Authors:** Susanna Daniel Fodeke, Elizabeth Ayuba Musa, Iliya Ishaya

**Affiliations:** 1 Department of Pharmaceutical Services, Jos University Teaching Hospital, Jos, Nigeria; 2 Department of Community Medicine, Ahmadu Bello University, Zaria, Nigeria

## Abstract

**Objective:**

To evaluate antibiotic prescribing practices in a Nigerian tertiary hospital using World Health Organization (WHO)-recommended process measures for Antimicrobial Stewardship (AMS).

**Design:**

Retrospective cross-sectional study. Patient folders (n = 231) between July 2019 and July 2020 were reviewed using a standardized tool. Relevant data were extracted and analyzed descriptively using SPSS v23, after ethical approval.

**Setting:**

Jos University Teaching Hospital (JUTH), a tertiary hospital in Nigeria.

**Participants:**

A simple random sample of patients admitted to the male medical wards during the review period.

**Results:**

Antibiotics were prescribed in 178 cases (77.1%) but only 149 (64.5%) had a documented indication. Of the patients with no documented indication for antibiotics, 35.3% still received them, often justified as prophylaxis. Ceftriaxone (31.8%) and metronidazole (24.5%) were the most prescribed antibiotics. Correct posology was observed in 77.5% of prescriptions. Adherence to prescribed regimens was low (25.3%), with financial constraints and missed doses being major barriers. Antibiotic time-outs were observed in 34.3% of cases with only 24.7% cases shifting to definitive therapy based on culture results. Empirical therapy accounted for 75.3% of prescriptions.

**Conclusion:**

The study revealed suboptimal implementation of AMS principles, characterized by a high reliance on empirical therapy and poor adherence to guideline-based practices. This underscores the urgent need for institutional AMS programs to optimize antibiotic use in similar low- and middle-income country hospital settings.

## Introduction

Antibiotics remain one of the greatest discoveries of modern science. They were hailed as “magic bullets” that could target harmful microbes without harming the host.^1^ Since then, scientists have developed a wide range of antibiotics, from narrow-spectrum agents to broad-spectrum ones used in complex infections.^2^ However, over time, bacteria have caught up with the available antibiotics, evolving to resist these drugs, thereby making many treatments less effective or even useless.^3^ At the same time, new antibiotics aren’t being developed quickly enough to keep up with this resistance.^4^

This growing crisis, known as antimicrobial resistance (AMR), poses a significant threat to global health.^3^ It leads to longer illnesses, higher death rates, and increased healthcare costs, especially in low- and middle-income countries (LMICs) where access to newer or stronger antibiotics is limited.^5,6^

The World Health Assembly’s endorsement of the Global Action Plan on AMR in May 2015,^7^ and the Political Declaration of the High-Level Meeting of the General Assembly on AMR in September 2017,^8^ both recognize AMR as a global health threat, with misuse and overuse of antibiotics as key drivers. Studies show that this burden is disproportionately higher in LMICs where factors such as limited surveillance, inadequate infrastructure, and unregulated access to antibiotics worsen the problem.^9,10^ In response to this threat, Antimicrobial Stewardship (AMS) programs have emerged to combat AMR by promoting the rational use of antibiotics.^11^

The idea of AMS first gained traction in the 1990s through McGowan and Gerding, who argued for a more controlled, evidence-based approach to antibiotic use, considering the long-term consequences of inappropriate prescribing.^12^ Today, AMS is fully supported by the World Health Organization (WHO) and other international health organizations.^13,14^ Even so, estimates show that up to half of all hospital antibiotic prescriptions may be inappropriate.^15,16^

One of the practical tools within AMS is the “MINDME” guideline,^17^ which reminds prescribers that:

- **M**icrobiology guides therapy
- **I**ndications should be evidence-based
- **N**arrowest effective spectrum should be used
- **D**osage suited to site or type of infection should be used
- **M**inimize duration
- **E**nsure monotherapy where possible

Beyond the role of prescribers, a multidisciplinary approach involving prescribers, pharmacists, microbiologists, and infection control personnel is highly beneficial to the success of AMS, as it enhances clinical decision-making, ensures accountability, and fosters collaboration across departments.^18^ AMS efforts also increasingly rely on process measures, such as documentation of indication, dose accuracy, and review of therapy, as practical frameworks for assessing prescribing quality.^18,19^

Pharmacists have an essential and expanding role to contribute to AMS, both within hospital and community settings. As highlighted in recent commentary, pharmacists are uniquely positioned to promote rational antimicrobial use through activities such as optimizing prescriptions, ensuring adherence to guidelines, educating healthcare providers and patients, and monitoring antimicrobial utilization trends.^20^ A 20-year systematic review further documents the evolution of this role, noting that pharmacists have progressively moved from primarily dispensing functions to active participation in multidisciplinary AMS teams, policy development, and leadership of stewardship interventions that improve patient outcomes and reduce AMR risk.^21^ Consequently, pharmacists must ensure they are not relegated to the sidelines by proactively seeking active involvement in AMS activities. By leveraging their expertise, they can help close key gaps in stewardship, including optimizing prescribing practices, improving adherence, and facilitating timely transitions to definitive therapy.

Despite growing attention to stewardship globally, gaps in AMS implementation remain, particularly in LMICs where data remains limited and systemic challenges persist.^5^ This study aimed to assess antibiotic use practices in a Nigerian tertiary hospital using WHO-recommended process indicators.^22^

## Methods

### Study design and setting

This was a retrospective, cross-sectional study conducted in the male medical wards (wards 10 and 11) of Jos University Teaching Hospital (JUTH), Nigeria. JUTH is a major tertiary healthcare facility in North Central Nigeria.^23^ Data was collected from patient medical records spanning July 2019 to July 2020.

### Study population and sampling

Eligible cases were defined as adult patients (≥18 years) admitted into the male medical wards of JUTH from July 2019 to July 2020, while those admitted outside this period or admitted to other wards were excluded. A simple random sampling method was used to select patients who met the inclusion criteria. While a sample size of 384 was initially calculated using the Kish formula,^24^ the final cohort of 231 cases represents all retrievable records available during the study period.

This shortfall resulted from COVID-19 restrictions which constrained access to hospital records due to limited records staff availability for supervised folder access. Consequently, only a limited number of records could be reviewed, and the study is presented as a descriptive baseline audit of stewardship process measures rather than a powered hypothesis-testing analysis.

### Data collection

A standardized data collection proforma, adapted from page 52 of the WHO’s *AMS Programs in Health-Care Facilities in LMICs: A Practical Toolkit*, was used for data extraction.^22^ The proforma captured patient demographics, primary diagnosis, details of prescribed antibiotics (name, dose, route, duration, indication), relevant laboratory results (specifically microbial culture and sensitivity tests), and documentation of 48-hour antibiotic reviews. Cases with missing medical administration records (MARs) or missing test results were recorded as separate “missing” categories; no imputation was performed. Following WHO AMS toolkit criteria, generic “prophylaxis” labels without a specific diagnosis were categorized as irrational. Adherence to prescribed antibiotics was assessed exclusively for the duration of the inpatient stay via MAR review; no follow-up was conducted after patient discharge.

### Data analysis

Descriptive statistics, including frequencies and percentages, were used to summarize the collected data. All analyses were performed using Statistical Package for Social Sciences (SPSS) version 23.

## Results

**Figure 1.**
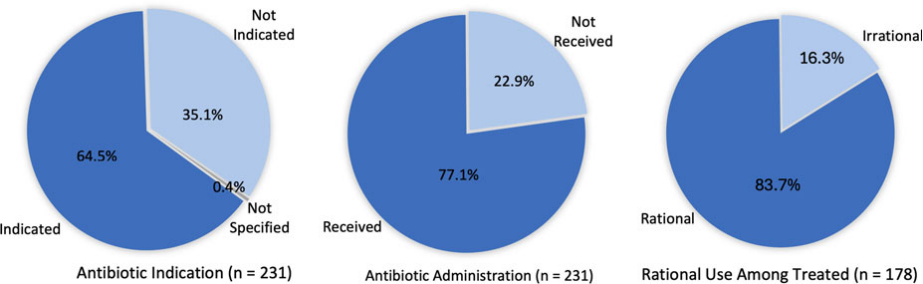
Overview of antibiotic indications and use among patients.

Figure 1 provides an overview of antibiotic indication and use among the 231 patient folders reviewed. A diagnosis warranting antibiotic therapy was documented in 149 cases (64.5%) while 81 patients (35.1%) had no such indication and one additional case lacked diagnosis details but still received antibiotics, bringing the number of patients without an antibiotic indication to 82. Despite this, 29 patients (35.3%) out of the 82 were still administered antibiotics, bringing the total number of patients who were administered antibiotics to 178 (77.1%). This indicates that antibiotics were rationally administered in only 83.7% of all treated cases while (16.3%) prescriptions were irrational.

**Figure 2.**
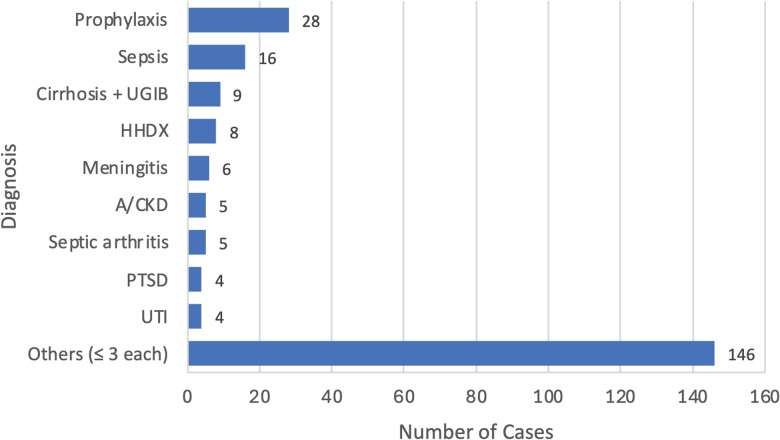
Diagnoses associated with antibiotic prescribing in the study population (n = 231). Other: diagnoses occurring ≤ 3 times (n = 146 patients). A detailed list of diagnoses included in the “Other” category is provided in Supplementary Table 1. Cirrhosis+UGIB, cirrhosis with upper gastrointestinal bleeding; HHDx, hypertensive heart disease; A/CKD, acute on chronic kidney disease; PTSD, post-traumatic stress disorder; UTI, urinary tract infection.

Figure 2 shows top diagnoses encountered within the sample size (n = 231), with prophylaxis being the most frequently indicated (28 cases, 12.1%), followed by sepsis (16 cases, 6.9%) and liver cirrhosis with upper gastrointestinal bleeding (9 cases, 3.9%). Per WHO process measure standards, prophylaxis was categorized as irrational because records lacked specific diagnostic justification. These 28 cases, plus one missing diagnosis, account for the 29-prescription gap between documented indications (n = 149) and total antibiotic use (n = 178). The “Other” category (≤3 occurrences; n = 146) comprised 50 distinct diagnoses grouped into 7 broad clinical categories. These included various respiratory infections, neurological disorders, organ diseases (renal, hepatic, and cardiovascular), localized infections, and other uncommon conditions. A detailed list of diagnoses included in the “Other” category is provided in Supplementary Table 1.

**Figure 3.**
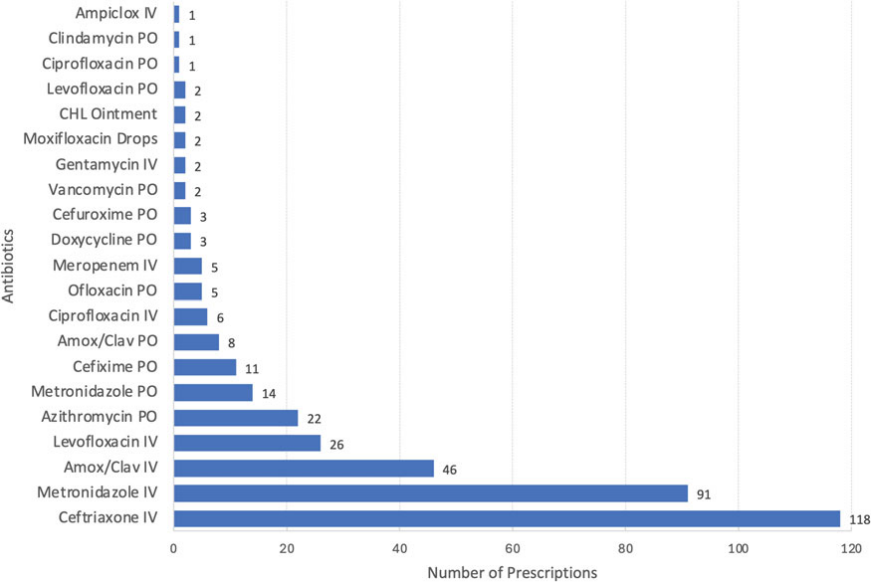
Antibiotic agents and routes of administration (n = 178). Amox/Clav, amoxicillin/Clavulanic acid; CHL, chloramphenicol; IV, intravenous; PO, oral.

Figure 3 presents an overview of all prescribed antibiotics. The most prescribed antibiotics were intravenous (IV) Ceftriaxone used in 118 cases (31.8%), IV Metronidazole in 91 cases (24.5%) and IV Amoxicillin/Clavulanate in 46 cases (12.4%), while the least prescribed agents were IV Ciprofloxacin, PO Clindamycin, and IV Amoxicillin (1 case each).

**Figure 4.**
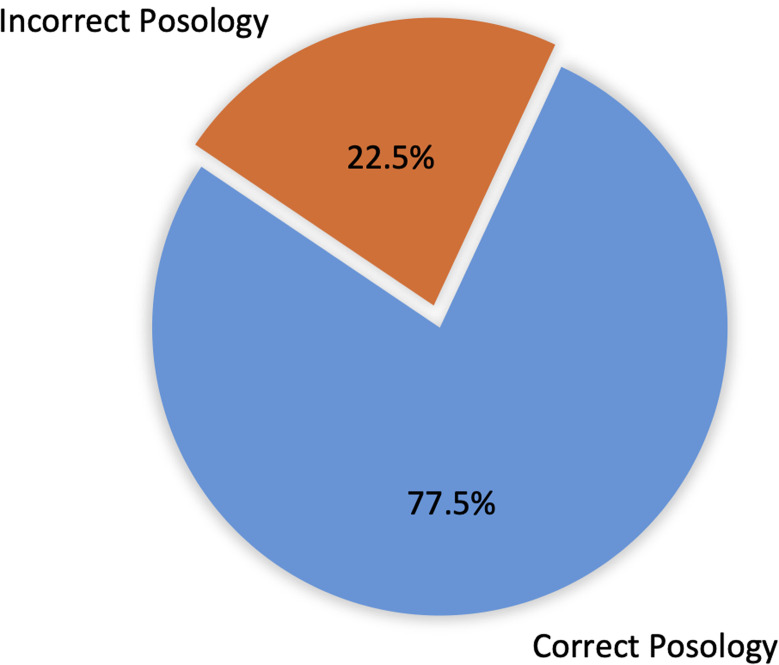
Quality of antibiotic prescriptions assessed by posology (dose, route, frequency) among reviewed cases (n = 178).

Figure 4 details the quality of total antibiotic prescriptions (n = 178) in terms of posology: correct dose, dosing frequency, and route of administration. Accurate posology was observed in 138 cases (77.5%) with the other 40 cases (22.5%) found lacking.

**Figure 5.**
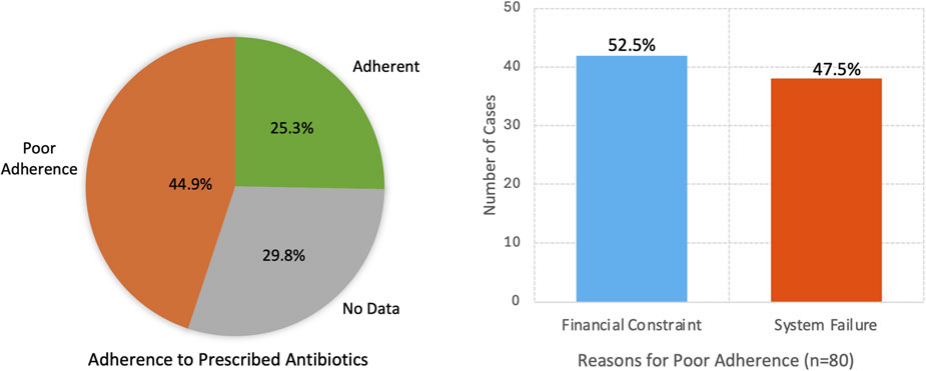
Adherence to prescribed antibiotics (n = 178) and documented reasons for poor adherence (n = 80).

Figure 5 reports adherence to prescribed antibiotic regimens (n = 178). Adherence was documented in 45 cases (25.3%), while poor adherence was observed in 80 cases (44.9%). In 53 cases (29.8%), medication administration records were missing from the patient’s folder. These cases were categorized separately to avoid miscalculation but may bias adherence estimates. Poor adherence to prescribed regimens (80 cases) was attributed to two (2) main factors identified in the patient records: patients’ inability to afford prescribed medications (financial constraint) in 42 cases (52.5%) and 38 cases of missed or unrecorded doses (47.5%).

**Figure 6.**
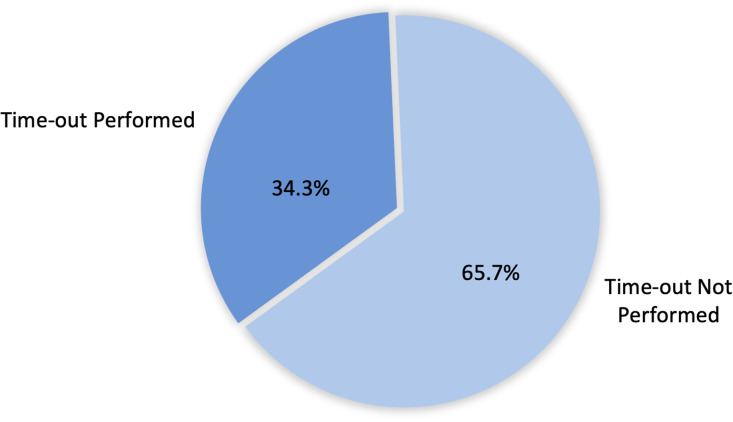
Frequency of 48-hour antibiotic reviews (“time-outs”) conducted among patients (n = 178).

Figure 6 illustrates our findings on the implementation of antibiotic time-outs, showing that 48-hour reviews to reassess therapy were conducted in only 61 cases (34.3%), while the remaining 117 cases (65.7%) lacked this critical AMS intervention.

**Figure 7.**
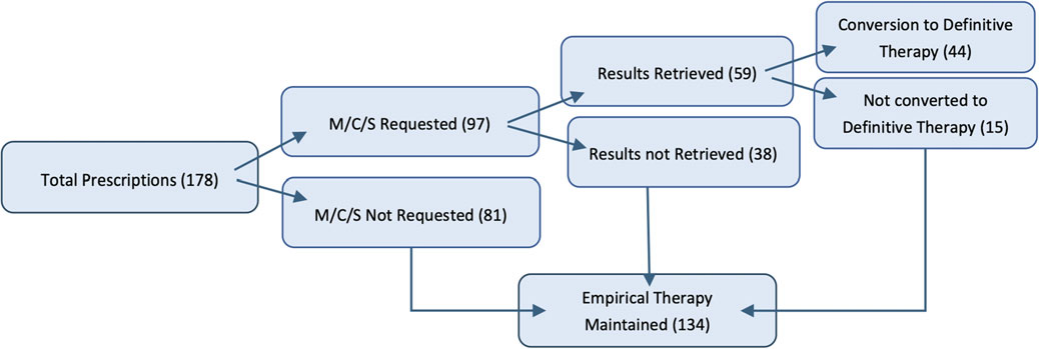
Utilization of microbial culture and sensitivity (M/C/S) testing and its influence on antibiotic prescribing (n = 178).

Figure 7 summarizes Microbial Culture and Sensitivity Testing (M/C/S) utilization. M/C/S was requested in 97 cases (54.5%) but not in 81 cases (45.5%). Of the requested tests, results were retrieved in only 59 cases (60.8%). Ultimately, only 44 cases (24.7% of total antibiotic prescriptions) were adjusted to definitive therapy, while the remaining 134 cases (75.3%) continued empirically.

## Discussion

This retrospective cross-sectional study explored AMS practices within the male medical wards of a major tertiary healthcare center in Nigeria. It assessed the use of process measures as an important AMS tool which plays a key role in curbing the threat of AMR.

Our findings demonstrate suboptimal adherence to AMS principles, which aligns with challenges commonly observed in LMIC settings.^25^ The high empirical prescribing rate (75.3%) highlights systemic and diagnostic limitations, including inadequate access to timely microbial culture testing and poor clinical documentation. This reliance on empirical therapy, coupled with the low rate of transition to definitive therapy based on culture results (24.7%), poses a substantial risk for treatment failures and the propagation of AMR. To support this finding, Alanazi et al. reported that limited adoption of antibiotic de-escalation was associated with increased antibiotic consumption, longer hospital stays, and higher costs, highlighting the importance of timely culture-guided adjustments.^26^

In Figure 2, where diagnoses were highlighted, it is not surprising that prophylaxis emerged as the most frequent indication, as many other studies have reported similar findings. For instance, in a global pediatric survey, almost 29% of antibiotic prescriptions were for prophylaxis (primarily medical prophylaxis).^27^ In a tertiary hospital in Malaysia, prophylactic use accounted for 11.5% of all prescriptions, more than any diagnosis-driven empiric therapy.^28^ PostCOVID-19 point-prevalence surveys in India and Pakistan also found prophylaxis rates ranging from 37% to 85%, especially in surgical settings.^29^ Prophylactic antibiotic use is a well-established practice, particularly in settings where the risk of hospital-acquired infections increases with length of stay.^30^ However, while prophylaxis plays a valuable role in infection prevention, it must be applied judiciously and in line with clinical guidelines to avoid contributing to AMR.^31^

The predominance of broad-spectrum antibiotics like ceftriaxone and metronidazole is influenced by the high rate of empirical antibiotic therapy, and as discussed, can worsen the emergence of AMR. These findings underscore the need for targeted interventions to promote narrower-spectrum agent use where appropriate, in line with international guidelines.^32–34^

Although our study showed that 77.5% of prescriptions had correct posology (dose, frequency, route), it remains essential that both prescribing and administration practices are rational and adhere strictly to established guidelines.

The observed low adherence to crucial process measures—such as poor documentation of indications for antibiotic use, infrequent conduct of 48-hour antibiotic time-outs (only 34.3%) and limited conversion from empirical to definitive therapy following microbial testing—indicates a significant gap in the implementation and monitoring of AMS activities.^35^ These deficiencies, alongside the apparent lack of formal AMS oversight, are likely compounded by broader systemic barriers such as patients’ out-of-pocket payment models and intermittent antibiotic stock-outs, which discourage culture testing and continuity of therapy. Together, these factors contribute to irrational prescribing patterns and hinder efforts to optimize antibiotic use within the hospital. These findings are consistent with previous studies conducted in similar resource-constrained environments that highlight the widespread challenge of inappropriate antibiotic prescribing and poor adherence to stewardship guidelines.^36,37^

While this study offers important insights, certain limitations should be considered when interpreting the findings. First, the sample size was smaller than the number estimated by the Kish formula, which may have reduced the representativeness and generalizability of the findings. Second, the study was restricted to male medical wards only. This decision reflected feasibility constraints during the COVID-19 period, when access to hospital records was already limited. While this focused approach provided valuable insights as a case study, it also limited the applicability of findings to other patient populations, such as female, pediatric, or surgical wards. Third, as a retrospective study, it relied heavily on the quality and completeness of patient medical records, which were sometimes inconsistent due to poor documentation. In addition, the data collection period (July 2019–July 2020) coincided with the early phase of the COVID-19 pandemic, which may have influenced healthcare practices and documentation due to activity restrictions. Gutema and Homa reported similar widespread disruptions to AMS and diagnostic activities during this period in African healthcare settings.^38^ Collectively, these limitations may impact the generalizability of our findings beyond the pandemic period and to other departments or hospitals without further investigation.

Despite these limitations, the study has notable strengths. First, by applying WHO-recommended process indicators, our findings can be directly compared with similar studies across LMICs, adding to the global evidence base. Second, it provides baseline institutional data for JUTH, which can inform tailored AMS interventions. To our knowledge, few Nigerian hospital studies have explicitly applied the WHO LMIC AMS process indicators; most prior work has used Global-PPS frameworks capturing related measures (eg, a multicenter PPS across northern Nigeria documented comparable process metrics).^39^ Our study therefore adds ward-level detail and quantifies gaps in documentation, 48-hour reviews, and culture-guided therapy practices in a Nigerian tertiary hospital setting. Furthermore, the persistent challenges observed in the study reflect real systemic barriers to effective AMS implementation in LMIC hospitals and emphasize the urgent need for comprehensive AMS programs in this and other Nigerian hospitals.

Given the gaps observed in prescribing practices and stewardship processes, several critical steps are needed to foster rational antibiotic use and mitigate the rise in AMR. These include establishment of active multidisciplinary AMS committees, enhanced healthcare provider education, improved documentation standards (potentially leveraging digital solutions), and increasing the accessibility and affordability of microbial diagnostics. These measures have been found to improve patient outcomes and drive AMS, thereby contributing to curbing the spread of AMR.^40^ Ultimately, the findings from this study should serve as a catalyst for institutional policy change, guiding the establishment of sustainable AMS structures, enhancing diagnostic capacity, and fostering accountability in antibiotic use across Nigerian tertiary hospitals. By addressing these gaps through structured AMS programs and stronger multidisciplinary engagement, hospitals like JUTH can not only optimize antibiotic use but also strengthen global efforts against AMR in resource-limited settings.

## Supporting information

10.1017/ash.2026.10343.sm001 Fodeke et al. supplementary material Fodeke et al. supplementary material

## Data Availability

De-identified data used in this study are available from the corresponding author upon request.

## Supplementary material

The supplementary material for this article can be found at https://doi.org/10.1017/ash.2026.10343.
