# Supplementary material for: Assessment of Antimicrobial Stewardship practices using process measures in a Nigerian tertiary hospital: a retrospective study
Source: Antimicrob Steward Healthc Epidemiol. 2026 Mar 27;6(1):e75. doi: 10.1017/ash.2026.10343 (PMC13104571; doi:10.1017/ash.2026.10343)
Supplement: Fodeke et al. supplementary material [file S2732494X2610343Xsup001.pdf]

Supplementary Table 1. Clinical conditions included in the 'Other' category of Figure 2
This table lists the 50 specific diagnoses included in the 'Other' category, organized under 7 broad clinical groups: Respiratory, Neurological, Cardiovascular, Renal/Urogenital, Gastrointestinal/Hepatic, Endocrine/Metabolic, and Infectious/General.

BROAD CLINICAL GROUP	SPECIFIC DIAGNOSES INCLUDED
**RESPIRATORY**	Community-acquired pneumonia, Hospital-acquired pneumonia, Pneumonitis, Pulmonary tuberculosis, Pulmonary tuberculosis rule-out pneumonia, Bilateral parenchymal disease, Acute chest syndrome
**NEUROLOGICAL**	Stroke/Cerebrovascular accident, Hemispheric stroke, Left hemiparesis, Left hemisphere stroke, Transient ischemic attack, Dementia, HIV-associated neurocognitive disorder, Psychotic disorder, Seizure disorder, Tinnitus
**CARDIOVASCULAR**	Congestive heart failure, Breathlessness due to congestive heart failure, Congestive heart failure secondary to dilated cardiomyopathy or hypertensive heart disease, Deep vein thrombosis, Hypertensive encephalopathy
**RENAL/UROGENITAL**	Acute kidney injury, Acute kidney injury secondary to lower urinary tract symptoms and gastroenteritis, Chronic kidney disease, End-stage renal disease, Complicated urinary tract infection, Posterior urethral valve/Pelvic Fracture Urethral Distraction Defect, Uropathy
**GASTROINTESTINAL/HEPATIC**	Chronic liver disease, Decompensated chronic liver disease, Hepatic encephalopathy, Viral hepatitis, Enteric fever, Gastritis, Epigastric pain, Diarrhea with abscess
**ENDOCRINE/METABOLIC**	Type 2 diabetes mellitus
**INFECTIOUS/GENERAL**	Bacterial viral hemorrhagic fever, Chronic hepatitis B with disseminated tuberculosis, Gonococcal infection, Sepsis of various origins, Wound infection, Foot gangrene, Leg ulcers, Right foot ulcer, Right thigh abscess, Stevens–Johnson syndrome, Trauma/Polytrauma, Eye injury with traumatic brain injury
